# Survivin as a Potential Mediator to Support Autoreactive Cell Survival in Myasthenia Gravis: A Human and Animal Model Study

**DOI:** 10.1371/journal.pone.0102231

**Published:** 2014-07-22

**Authors:** Linda L. Kusner, Michael J. Ciesielski, Alexander Marx, Henry J. Kaminski, Robert A. Fenstermaker

**Affiliations:** 1 Department of Pharmacology and Physiology, George Washington University, Washington, District of Columbia, United States of America; 2 Department of Neurology, George Washington University, Washington, District of Columbia, United States of America; 3 Department of Neurosurgery, Roswell Park Cancer Institute, Buffalo, New York, United States of America; 4 Center for Immunotherapy, Roswell Park Cancer Institute, Buffalo, New York, United States of America; 5 Institute of Pathology, University Medical Centre Mannheim, University of Heidelberg, Mannheim, Germany; University of Sydney, Australia

## Abstract

The mechanisms that underlie the development and maintenance of autoimmunity in myasthenia gravis are poorly understood. In this investigation, we evaluate the role of survivin, a member of the inhibitor of apoptosis protein family, in humans and in two animal models. We identified survivin expression in cells with B lymphocyte and plasma cells markers, and in the thymuses of patients with myasthenia gravis. A portion of survivin-expressing cells specifically bound a peptide derived from the alpha subunit of acetylcholine receptor indicating that they recognize the peptide. Thymuses of patients with myasthenia gravis had large numbers of survivin-positive cells with fewer cells in the thymuses of corticosteroid-treated patients. Application of a survivin vaccination strategy in mouse and rat models of myasthenia gravis demonstrated improved motor assessment, a reduction in acetylcholine receptor specific autoantibodies, and a retention of acetylcholine receptor at the neuromuscular junction, associated with marked reduction of survivin-expressing circulating CD20+ cells. These data strongly suggest that survivin expression in cells with lymphocyte and plasma cell markers occurs in patients with myasthenia gravis and in two animal models of myasthenia gravis. Survivin expression may be part of a mechanism that inhibits the apoptosis of autoreactive B cells in myasthenia gravis and other autoimmune disorders.

## Introduction

A central question in the study of autoimmune disorders concerns the mechanisms that maintain the persistence of autoreactive cells that produce disease. Myasthenia gravis (MG) is a T cell dependent, B cell mediated autoimmune disease in which antibodies are directed against proteins concentrated at the neuromuscular junction (NMJ), primarily the nicotinic acetylcholine receptor (AChR) [1]. The AChR antibody synthesis is dependent on autoantigen specific B cells. We hypothesized that certain inhibitor of apoptosis proteins (IAPs) that are expressed in neoplastic diseases might also support the pathologic survival of autoreactive immune cells.

IAPs are a family of proteins that contain one or more baculovirus repeat (BIR) domains originally found to influence apoptosis and restrict the activation of caspases, preventing the cell from undergoing cell death, specifically in cancers [2]. A prime candidate of the maintenance of autoreactive immune cells is survivin, a 16.5 kDa intracellular protein that belongs to the IAP family and contains one BIR domain [3]. Survivin is highly expressed during fetal development and is absent in normal adult tissue [4]. It interacts with the mitotic spindle and regulates cell division [5]–[7]. Furthermore, survivin's role as an anti-apoptotic protein is to modulates the function of a number of terminal effector cell death proteases (caspases) leading to inhibition of apoptosis [8]–[10]. These and other actions may account survivin's role in preventing the death of malignant cells [11]. Survivin has been implicated in the pathophysiology of certain autoimmune disorders [12], [13]. Survivin expression is significantly higher in the synovial tissue of rheumatoid arthritis (RA) patients with destructive disease and lower among patients receiving therapy with disease-modifying drugs, [13], [14] making *in situ* survivin expression an independent prognostic parameter for erosive RA. Circulating survivin is positively correlated with severity of juvenile RA [15]. Therefore, survivin expression appears to predict the clinical course of RA [16]; whereas, anti-survivin antibodies (a proxy for the anti-survivin immune response) are associated with less severe disease [13]. Survivin levels are also elevated in T cells and brain tissue of patients with primary progressive multiple sclerosis (MS), suggesting a distinct pathological function of surviving [17]. Mitogen-stimulated T lymphocytes from patients with active MS display high-level expression of survivin compared to patients with stable disease [18]. These observations indicate that survivin expression may be a common characteristic of autoimmunity and that it is likely to play a direct pathophysiologic role.

Survivin is expressed in many cancers and a number of studies have demonstrated that cancer patients have anti-survivin antibodies as well as T cells capable of recognizing survivin epitopes via interaction with MHC class I molecules on tumor cells. Cytotoxic T lymphocytes (CTL) recognize the MHC-class I molecule in complex with survivin epitopes eliciting a cytotoxic antitumor response [19]. Anti-tumor vaccine strategies have been used successfully to target tumor cells that express such cell-surface complexes [20]. One such strategy has employed a survivin peptide mimic (SVN53-67/M57), which is naturally processed into epitopes that are presented by human MHC class I molecules, as well as by murine H2-Kb molecules (as a model of the human response) [21]–[23]. The SVN53-67/M57 peptide mimic contains a core CTL epitope SVN56-64 modified by substitution of methionine for cysteine at amino acid position 57. This core epitope binds to HLA-A*02 molecules with higher affinity than the wild type molecule producing a potent immune response which is cross-reactive to the wild type molecule [22]. Conjugation of the peptide to Keyhole Limpet Hemocyanin (KLH) to form SVN53-67/M57-KLH provides enhanced stimulation of the immune system *in vivo*. The SVN53-67/M57-KLH vaccine produces CTL-mediated killing of many different survivin-expressing cancer cell types, including glioma and B cell lymphoma [21]. SVN53-67/M57-KLH is currently in clinical trials as a cancer immunotherapeutic.

We chose MG as a disease to begin to address whether survivin is involved in the pathophysiology of antibody-mediated autoimmunity. MG is among the best characterized antibody-mediated diseases with the primary antigenic targets being neuromuscular junction (NMJ) proteins, primarily the nicotinic acetylcholine receptor (AChR) [1], [24]. The antibodies produce a reduction of AChR number and damage the muscle endplate, leading to a failure of neuromuscular transmission with resulting muscle weakness. The AChR antibodies of patients are polyclonal and recognize a complex repertoire of epitopes that differ among individual patients. Experimentally acquired myasthenia gravis (EAMG) produced by administration of purified AChR to rodents has similar immune characteristics to the human disease [25], [26]. Studies that highlight the mechanisms involved in driving the autoimmune process have largely focused on the complexity of the breakdown of tolerance [27] but have not addressed in detail the mechanisms by which autoreactive B cells may escape programmed cell death.

In the present investigation, we assessed survivin expression in various subsets of peripheral blood mononuclear cells (PBMC) of patients with MG. Having identified survivin expression in cells that co-express certain plasma cell and B cell phenotypic markers, we explored the effects of specific survivin peptide vaccination on moderation of active EAMG in two rodent models.

## Methods

### Ethics statement for human subjects

Blood specimens were collected from patients of the Department of Neurology at George Washington University. For patients with MG, entrance criteria for participation were: 1) clinical diagnosis of MG; 2) age ≥18 years of age; 3) confirmatory diagnostic test including positive tensilon, or ice pack test, or repetitive decrement on repeated stimulation EMG, or single fiber examination demonstrating a neuromuscular defect, or presence of serum acetylcholine receptor, or muscle specific kinase antibodies; and, 4) willingness to participate and ability to provide informed consent. Exclusion criterion was limited to inability to provide informed consent. The MG Foundation of America Clinical Research Standards were applied for subject classification [28]. Control subject inclusion criteria were limited to willingness to participate and ability to provide informed consent. Control subject exclusion criteria were age ≤18 years of age and treatment with prednisone or any immunosuppressive or immune modulator in the previous 12 months. All participants provided written consent for inclusion in the study. The study was approved by the George Washington University Institutional Review Board.(Table 1) Deidentified thymic samples were obtained and analyzed by the Institute of Pathology, University Medical Centre Mannheim with the approval (#2013-802RA-MA) of the Ethics Committee of the University.

**Table 1:** Demographic and treatment characteristics of myasthenic patients (1–15) and controls (16–25) consisting of individuals with non-myasthenic neurologic conditions.

Patient	Sex	Age	MGFA class (Diag/Draw)	AChR level nmol/L	Therapy	Other conditions	Symptom Duration
1	F	78	2a/2a	10	prednisone	-	3 yrs
2	F	19	1/1	2.7	none	-	6 mos
3	M	62	1/1	11.7	none	-	ND
4	F	42	1/1	6.9	none	-	2 mos
5	F	20	2/2	3.76	prednisone,pyridostigmine	-	4 mos
6	M	62	2b/2b	0.86	prednisone,pyridostigmine	-	4 mos
7	F	20	3/remission	30	prednisone	-	4 yrs
8	F	74	1/remission	68	prednisone	-	7 mos
9	M	61	2a/2a	5.09	prednisone, cellcept	-	4 mos
10	M	61	1/1	1.9	prednisone	-	1.5 mos
11	M	57	1/remission	22.6	prednisone	-	6 mos
12	F	53	2/remission	33/0	prednisone, cellcept	Type I DM	3 yrs
13	M	66	2b/2b	10	none	-	2.5 yrs
14	M	55	1/1	9.5	pyridostigmine	-	2 mos
15	F	57	2b/2b	0	none	Rheumatoid Arthritis	9 mos
16	M	53		ND	none	Uveitis	
17	F	48		ND	none	Hypothyroid	
18	F	38		ND	none	-	
19	F	34		ND	none	Cervical Spondylosis	
20	F	35		ND	none	-	
21	M	38		ND	none	Tremor	
22	M	54		ND	none	Neck Pain	
23	M	54		ND	none	Meningioma	
24	F	24		ND	none	Migraine	
25	F	70		ND	none	Hearing Problems	

### Analysis of MG patients and controls by flow cytometry (FACS)

Whole blood samples were used to ensure inclusion of all potential immune cells that may express survivin. Blood was diluted in cell staining buffer containing flow cytometry staining buffer (PBS/1% FCS, cat 420201 BioLegend, San Diego, CA) and TruStain fcX-Blocking reagent (cat 422302, BioLegend). Specific direct-labeled antibodies to cell surface antigens: CD8 (clone SFCI21Thy2D3 (T8) FITC, Beckman-Coulter, Brea, CA); CD20 (clone 2H7 PE and/or PE/Cy7, BioLegend); CD27 (clone M-T271 PE, BioLegend); CD38 (clone HIT-2 APC/Cy7, BioLegend); CD138 (clone DL-101 PerCP/Cy5.5, BioLegend) was added. After washes in PBS, cells were incubated in RBC lysis combination 1% paraformaldehyde fixation buffer (cat 422401 BioLegend). Following primary fixation cells were resuspended in permeabilization buffer (cat 421002 BioLegend). Survivin antibody (clone 60.11 DyLight650, Novus Biologicals, Littleton, CO) was added. Cells were washed in permeabilization buffer (cat 421002 BioLegend) followed with a final PBS wash and resuspension in Fluorofix fixation buffer (cat 422101, BioLegend). Sample acquisition was obtained on a Core facility BD LSR Fortessa 3 laser flow cytometer with 11 color detection and separate interrogation of sample for each laser. Samples were acquired with FACSDiva software followed with analysis using FCS Express software. Analysis was based upon isolated gating of lymphocyte populations and co-localization of survivin with specific CD markers as indicated. Experiments utilizing Acetylcholine Receptor Peptide (AChR) were performed as above with the addition of FITC-labeled recombinant AChR alpha 1 subunit (H00001134-Q01, peptide: 146–232 amino acid, Novus Biologicals, Littleton, CO) to the cell surface label cocktail.

### Survivin staining in the thymus

Eleven thymuses from MG patients were analyzed for survivin expression. All patients were AChR antibody positive. The treated group (5 with immunosuppressive agents and 2 with prednisolone alone) consisted of 6 women, 1 man, age range 18–42 years, mean 27 years). The untreated group had received no immunosuppressives or corticosteroids and consisted of 3 women, 1 man, age range 13–27 years, mean 22 years. Duration of symptoms prior to surgery was not documented for the majority of patients. Survivin expression was detected in neutral, buffered, formalin-fixed, paraffin-embedded human thymic tissue by immunohistochemistry as described [29] using the following reagents and methods: antigen retrieval in Novocastra antigen retrieval solution pH 9.0 (Leica, RE7119); anti-survivin monoclonal rabbit antibody (clone EP2880Y) (ab76424, Abcam Inc., Cambridge, MA, USA); blocking of endogenous peroxidase (DAKO blocking solution, S2013); detection of bound anti-survivin antibodies by the immunoperoxidase/DAB-based DAKO REAL detection system (K5007, DAKO GmbH, Hamburg, Germany).

### Ethics statement for animal use

Six- to eight-week-old male C57BL/6J mice (Jackson Laboratories, Bar Harbor, ME) and 100–124 gram female Lewis rats (Harlan, Indianapolis, IN) were used for the study. Animals were maintained in the George Washington University animal facility. The GWU animal facility follows IACUC, AAALAT, and AALAS standards concerning appropriate housing, cage cleaning procedure, air purity, feed, temperature, humidity, light and dark cycle. Animals were housed in isolator cages in a pathogen-free environment, and rodent chow and water were provided ad libitum. A veterinarian is on staff and observed the health of the animals throughout the study. All animal studies were conducted according to protocol approved by the George Washington University Institutional Animal Care and Use Committee (Permit No. A247). We used the reporting standards set by the NINDS for all outcome measurements [30].

### Post-treatment with SVN53-67/M57-KLH in active EAMG

AChR was purified from the electric organs of the *Torpedo californica* by affinity chromatography [31]. Nineteen male C57BL/6J mice were injected subcutaneously (s.c.), two injections at the shoulders and two injections at the base of tail, with *Torpedo* AChR (tAChR, 40 µg) in complete Freunds' adjuvant (Sigma) on day 0, day 24, and 52. Three s.c. injections of 100 µg SVN53-67/M57-KLH (nine mice) or PBS (ten mice) in incomplete Freunds' adjuvant (IFA, Sigma) were given at weekly intervals starting on day 42. The last two injections of SVN53-67/M57-KLH were given on days 59 and 73. Mice were evaluated in a blinded fashion biweekly using the generally accepted motor strength scale [32]. Clinical severity scale (1–4) was used: 0 =  no weakness observed, 1 =  slight weakness after exercise, 2 =  initial weakness, 3 =  weakness plus respiratory difficulty, and 4 =  moribund. Grip strength and body weight measurements were assessed. A grip strength meter (Columbus Instruments, Columbus, OH) with digital force gauge (Ametek, Largo, FL) was used to assess the peak force by which an animal grasped a grid pull bar. Each mouse was exercised before measurements were taken. Mean grip strength (N) from five pulls for each mouse was used for analysis. Grip strength measurements were given as a mean value for each group/week. Animals were sacrificed on day 80.

### Pre-treatment with SVN53-67/M57-KLH in active EAMG

We evaluated pre-treatment with SVN53-67/M57-KLH for efficacy in a rat model of EAMG. Pre-treatment with SVN53-67/M57-KLH involved three s.c. injections of 100 µg SVN53-67/M57-KLH in incomplete Freunds' adjuvant (IFA, Sigma) given at weekly intervals to eight rats, and eight rats received PBS in IFA. Active EAMG was produced a week after final SVN53-67/M57-KLH injection. Sixteen rats total received s.c. injection of 40 µg tAChR in complete Freunds' adjuvant (Sigma) given in two injections at the base of the tail. Rats were evaluated in a blinded fashion using the generally accepted motor strength scale [32], and body weight measurement. Grip strength was performed on all animals with twenty pulls before five measurements were recorded. Animals were sacrificed 47 days after AChR injection.

### Isolation of splenic cells from SVN53-67/M57-KLH and PBS treated EAMG mice

Spleens were harvested from EAMG induced animals and cells were teased from mouse spleens with sterile forceps and passed through a 70 µm filters (BD Falcon) in DMEM. Splenocytes were centrifuged and the pellet was re-suspended in RBC lysis buffer (R&D Systems). Cells were washed twice and re-suspended in complete RPMI culture medium. Splenocytes were frozen in DMEM containing 10% fetal bovine serum and 10% DMSO [33].

### Analysis of splenocyte populations for survivin expression

Specific direct labeled antibody to cell surface antigen CD20 (BioLegend, San Diego, CA) was used to assess splenocyte population. Following primary fixation in Fluorofix fixation buffer (cat 422101, BioLegend) cells were resuspended in permeabilization buffer (cat 421002, BioLegend). Survivin antibody (clone 60.11 FITC, Novus Biologicals, Littleton, CO) was added. Cells were washed in permeabilization buffer (cat 421002, BioLegend) followed with a final PBS wash and resuspension in Fluorofix fixation buffer (cat 422101, BioLegend). Sample acquisition was obtained on a Core facility BD FACSCalibur flow cytometer running CellQuest software followed with analysis using FCS express software. Analysis was based upon isolated gating of lymphocyte populations and co-localization of survivin with specific CD markers as indicated.

### Direct H2-Kb Tetramer Binding Assay

MHC class I tetramers specific to survivin peptide epitopes were used to assess the presence of survivin-specific T cells generated by survivin vaccine (SurVaxM) stimulation, as described above. PBMC from vaccinated animals were isolated following immunization with SurVaxM. Becton Dickinson MHC class I tetramers (Chicago, IL) were designed to recognize T cell receptors that bind the core CTL epitope of SurVaxM [21]. Survivin-specific R-PE-labeled tetramers were incubated with PBMC for 10 minutes at 25°C. Samples were incubated with anti-CD8 FITC (clone T8, Beckman Coulter, Brea, CA) for 20 minutes at 4°C. Cells were fixed for analysis by flow cytometry. CD8+ cells that were doubly labeled were considered positive for tetramer, ovalbumin loaded tetramers were utilized as a negative control. Data were analyzed using FCS Express software. Analysis was based upon gating on CD8+ T cells only.

### Antibody determination

96 well plates (Immulon, Fisher Scientific, Pittsburgh, PA) were coated with tAChR (10 µg/ml). Serum samples were diluted in two concentrations and added to the plate (rat serum dilution, IgG, 1,000; IgG1, 1∶50,000; IgG2b, 1∶1,000), (mouse serum dilution: IgG, 1∶20,000; IgG1, 1∶40,000; IgG2a, 1∶5,000; IgG2b, 1∶40,000). Washed plates were incubated with horseradish peroxidase conjugated antibodies: Anti-Rat IgG, Anti-Rat IgG1, or Anti-Rat IgG2b, Anti-Mouse IgG, Anti-Mouse IgG1, Anti-Mouse IgG2a, Anti-Mouse IgG2b (Alpha Diagnostics, San Antonio, TX). The color reaction was developed with SureBlue TMB peroxidase substrate (KPL, Gaithersburg, MD). Reaction was stopped with HCl and read at 450 nm on a Varioskan Flash microplate reader (Thermo Scientific, Waltham, MA). Samples and controls were analyzed in triplicates.

### Comparison of AChR content at the NMJ by Alexa Fluor 594 –bungarotoxin assessment

Ten micron cryosections of diaphragms were stained with Alexa Fluor 594 -bungarotoxin (Life Technologies, Grand Island, NY). The sections were viewed with Leica DFC310 FX and digital images captured with the Leica microsystems LAS AF6000 modular systems (Wetzlar, Germany) and analyzed with Image Pro software (Media Cybernetics, Inc., Rockville, MD). NMJs determined by fluorescence were defined as areas of interest. Pixel density measurements were determined and mean values obtained for each animal (n = 8/group for mouse study and n = 5/group for rat study) [34], [35].

### Statistical analysis

The data were analyzed and tested for statistical significance using paired t-tests and ANOVA. Results were considered significantly different when *P*<0.05.

## Results

### Survivin expression in peripheral blood mononuclear cells (PBMC)

**Figure 1:**
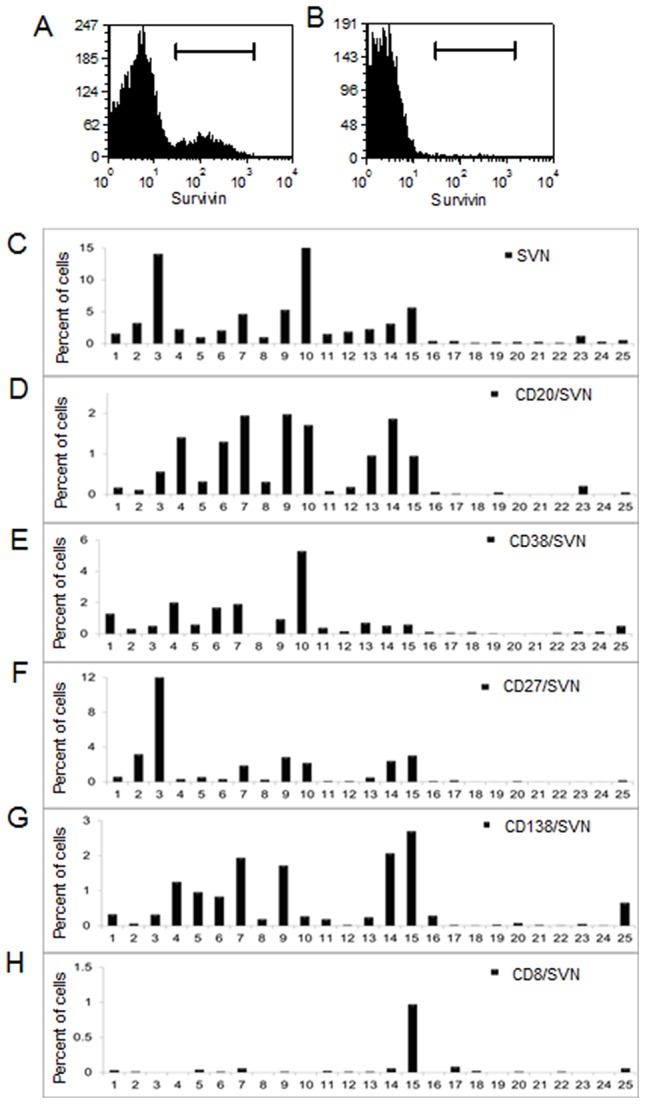
Survivin expression in myasthenia gravis patients (1–15) and control (16–25) PBMCs. **A,B**) Representative FACS analysis of total survivin expression of (A) myasthenic patient (7) and (B) control (24) PBMC. **C–H**) PBMC were stained with antibodies to indicated markers. Data reflect the percentage of total cells staining positive for: **C**) Survivin (SVN) (p = 0.010), **D**) CD20+/SVN+ (p = 0.0009), **E**) CD38+/SVN+ (p = 0.02), **F**) CD27+/SVN+ (p = 0.05), **G**) CD138+/SVN+ (p = 0.01) and **H**) CD8+/SVN+ (p = 0.4). Mean values of patients and controls were compared using two way ANOVA.

We examined survivin expression in patients with MG (*n* = 15) and control subjects (*n* = 10) with medical conditions indicated in Table 1. During the course of the investigation, no MuSK antibody patients were encountered and only one who was negative for both MuSK and AChR antibodies. No patient had a thymoma identified by imaging, and none had undergone a therapeutic thymectomy. There were no statistically significant differences in age (*P* = 0.28, ANOVA) or gender (*P* = 0.93, Chi Square) between the two groups (Table 1). MG patients had been treated with a range of medications including immunosuppressive therapies. Survivin expression in PBMC was assessed by flow cytometry (Fig. 1A, B, and C). MG patients consistently exhibited survivin-positive white blood cells (Fig. 1A and C); whereas, control patients did not (Fig. 1B and C). Cells were co-stained with anti-CD20 (Fig. 1D), CD27 (Fig. 1E), CD38 (Fig. 1F), CD138 (Fig. 1G), and CD8 (control) (Fig. 1H) markers. Survivin expression was significantly greater in PBMCs from MG patients and was strongly associated with CD20, CD27, CD38 and CD138 expression (Fig. 1C–G). In contrast there was no significant association between survivin and CD8 expression except for a single patient with both MG and rheumatoid arthritis who had high survivin levels in CD8+ cells (Fig. 1H). (Values for all datasets appear in Table S1.)

### Acetylcholine receptor alpha peptide binds to survivin positive cells

**Figure 2:**
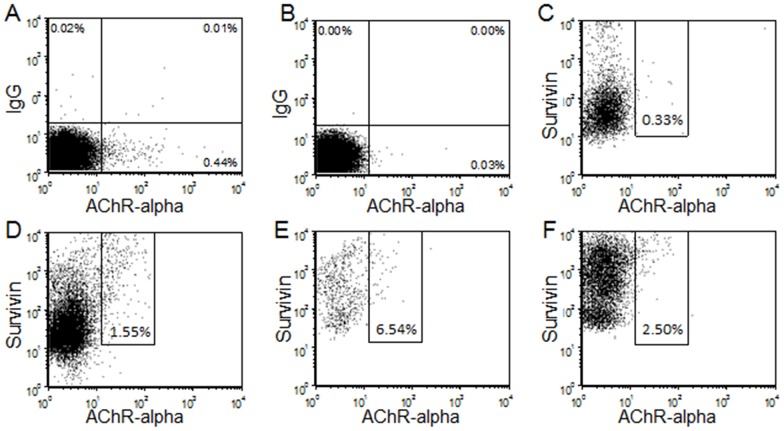
Acetylcholine receptor alpha peptide binding to survivin positive cells in myasthenics by five color FACS analysis. A) FITC labeled AChR-alpha subunit peptide binding to total myasthenic PBMC (0.44%), B) Excess unlabeled AChR-alpha subunit peptide followed by competitive binding of FITC-AChR peptide (0.03%). C) Control PBMC, subject number 19, (SVN+/CD38+/CD138+/CD20+ gated sub-population) showing FITC-AChR-alpha subunit peptide binding (0.33% of gated cells). D–F; Myasthenic patient PBMC (SVN+/CD38+/CD138+/CD20+ gated sub-population) showing FITC-AChR-alpha subunit peptide binding (1.55% (pt#10), 6.54% (pt#15) and 2.50% (pt#14) of total gated cells; *n* = 3).

A FITC-labeled AChR alpha 1 subunit peptide was tested for its ability to bind to PBMC from MG patients. Peptide binding was detected by flow cytometry and was competitively blocked with unlabeled AChR peptide indicating specificity of binding (Fig. 2A and B). AChR peptide bound to PBMC that were co-labeled with several B lymphocyte lineage and plasma cell markers (CD20, CD38 and CD138) in association with survivin expression. Figure 2C–F shows representative five color FACS analysis of PBMC gated on SVN+/CD38+/CD138+/CD20+ cells that also bind FITC-labeled AChR-alpha subunit peptide. FACS detected high numbers of CD20+/CD38+/CD138/SVN+ gated cells in patients with MG (Fig. 2D–F), but not in control subject (Fig. 2C).

### Thymus samples demonstrate survivin expression

**Figure 3:**
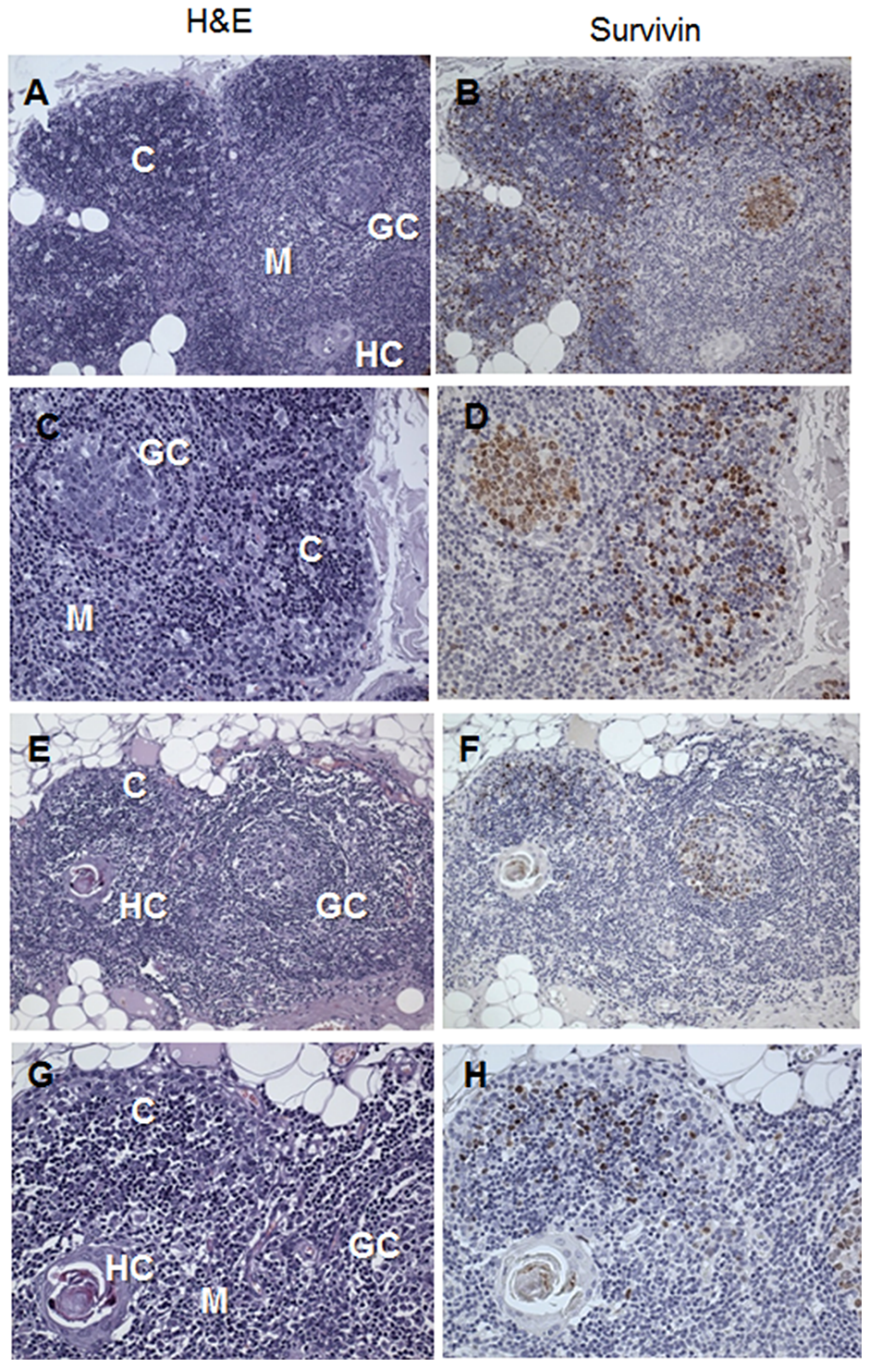
Survivin expression in the thymus. Representitive images from an analysis of eleven thymuses from MG patients were analyzed for survivin expression (immunosuppression treated, *n* = 7; immnosuppression naïve, *n* = 4). **A–D**) EOMG thymus from a 24-year-old woman who had clinical symptoms for 2 years and an AChR antibody level of 19.3 nmol and had never received immunosuppression or prednisolone showed a well developed cortex (C) and medulla (M), lymphofollicular hyperplasia with a germinal center (GC) close to Hassall's corpuscle (HC). **B,D**) High number of survivin positive cells in the cortex (C), cortico-medullary junction and the GC. **E–H**) EOMG thymus from a AChR antibody positive 21-year-old female with lymphofollicular hyperplasia after long-term immunosuppression showing cortical atrophy and a slightly regressive germinal center (GC) close to a Hassall's corpuscle (HC). **F,H**) Low number of survivin positive lymphocytes in the remnant cortical area (C), and the germinal center (GC) (A,B,E,F, ×100; C,D,G,H ×200).

Eighty percent of generalized myasthenia gravis patients with AChR positive antibody titers have thymic pathology [36]. We studied thymuses from eleven MG patients, 4 untreated and 7 treated with corticosteroids. The thymuses from the four untreated MG patients demonstrated lymphofollicular hyperplasia [37]. Cells in the cortex, cortico-medullary junction, and germinal centers were positive for survivin (Fig. 3A–D). The thymuses from patients that were treated with corticosteroids prior to thymectomy demonstrated fewer survivin-positive cells (Fig. 3E–H). In active germinal centers that persisted inside atrophic thymuses, a subpopulation of the germinal center cells remained survivin positive (Fig. 3F,H).

### Mouse model of EAMG demonstrates reduced weakness following SVN53-67/M57-KLH treatment

**Figure 4:**
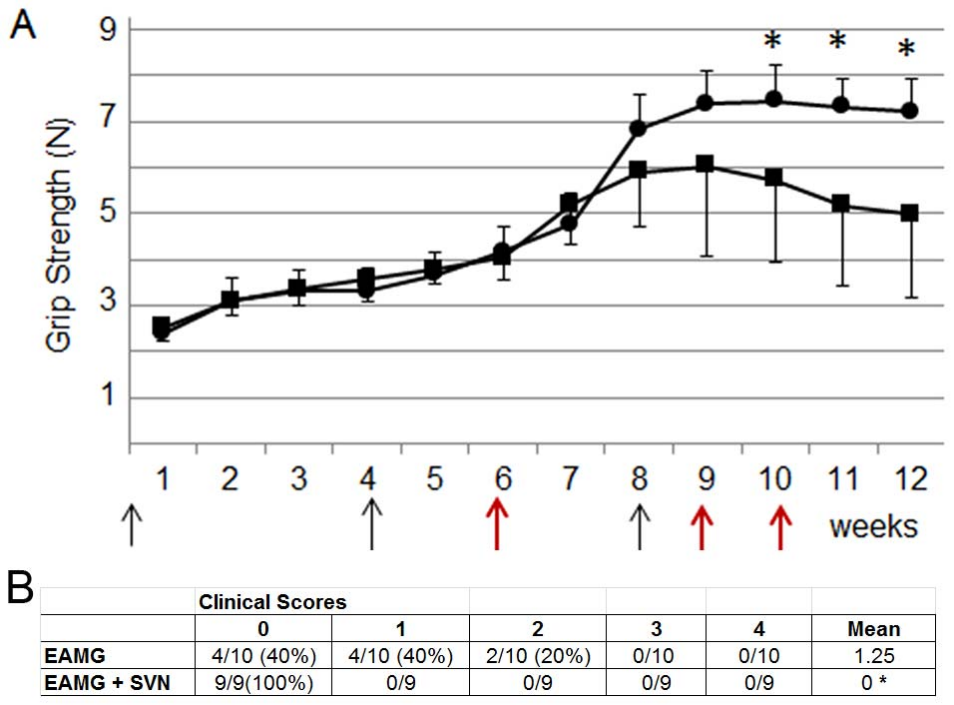
SVN53-67/M57-KLH post-treatment on active EAMG in the mouse model. **A**) Animals were injected with SVN53-67/M57-KLH at times marked by red arrows. Black arrows mark injection times of tAChR. Grip strength was monitored throughout experiment. Mice were treated with SVN53-67/M57-KLH (circles, *n* = 9), or PBS (squares, *n* = 10) (* denotes *P*<0.05, t-test). **B**) Table shows clinical scores of mice treated with SVN53-67/M57-KLH were significantly stronger than PBS treated mice (* denotes *P*<0.05, t-test).

Active EAMG was induced in mice by injections of torpedo AChR in complete Freund's adjuvant (CFA) on day 0, 24, and 52. Survivin vaccination was initiated on day 42, ten days prior to the last AChR injection and continued to day 73. Mice were evaluated for grip strength, weight and clinical scores weekly. SVN53-67/M57-KLH treatment (n = 9) demonstrated greater grip strength (statistically significant, *P*<0.05, t-test) starting at week 10 compared to PBS treatment controls (n = 10) (Fig. 4A, Table S2). Overall, mice treated with SVN53-67/M57-KLH did not show weakness and had a clinical score of zero. In contrast, 20% of mice treated with PBS demonstrated general weakness (inability to grip wire bar and limited motility, clinical score of 2) and 40% demonstrated weakness after exercise (clinical score of 1) (Fig. 4B).

### Survivin expression in EAMG mouse lymphocytes and effect of anti-survivin vaccine

**Figure 5:**
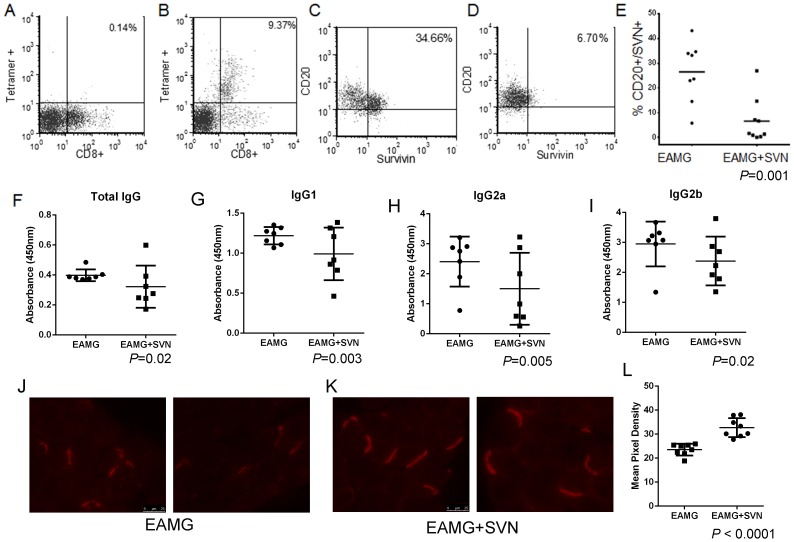
Analysis of post-treatment with SVN53-67/M57-KLH in EAMG mouse model. Survivin-specific iTag tetramer analysis of survivin-targeted CD8+ T cells in EAMG mice (**A**) PBS and (**B**) SVN53-67/M57-KLH-treatment. FACS analysis of PBMC with representative flow histograms of CD20+/SVN+ cells from (**C**) PBS and (**D**) SVN53-67/M57-KLH-treated mice. **E**) Mean values of groups were compared using two way ANOVA (*P* = 0.001). **F–I**) AChR specific total IgG (**F**), IgG1 (**G**), IgG2a (**H**) and IgG2b (**I**) levels were determined by ELISA on an AChR coated 96-well plate for PBS (*n* = 7) and SVN53-67/M57-KL (*n* = 7)-treated animals. Levels of total IgG, IgG1, IgG2a and IgG2b were significantly higher in the PBS treated group compared to SVN53-67/M57-KLH treated animals (*P* = 0.02 for total IgG and IgG2b; p<0.005 for IgG1 and IgG2a). **J–L**) AChR expression at the NMJ of PBS treated (*n* = 8) and SVN53-67/M57-KLH-treated (*n* = 8) mice was determined by imaging of Alexa Fluor 594 –bungarotoxin pixel density at the NMJ. Two representative images of PBS treated (**J**) and SVN53-67/M57-KLH-treated (**K**) mice are shown. Mean pixel density was determined (**L**) (*P* = 0.0001, t-test).

The survivin vaccine contains a number of class I CTL epitopes. Tetramer assays were performed to determine if the survivin vaccine induced production of survivin-specific CD8+ T cells. EAMG mice that were vaccinated with SVN53-67/M57-KLH exhibited an immune response that included larger numbers of survivin-specific CD8+ T cells (Fig. 5A, B). EAMG mice expressed survivin in CD20+ PBMC at high levels (range: 5.84%–43.14%; mean 26.5%) (Fig. 5C, E). In contrast, EAMG mice vaccinated with SVN53-67/M57-KLH had markedly reduced numbers of SVN+/CD20+ cells (range 0.10%–26.9%; mean 6.7%; *P* = 0.001) (Fig. 5D, E).

### AChR specific IgG subtypes were reduced in survivin vaccinated mice

ELISAs were performed to determine AChR specific total IgG, IgG1 and IgG2b (Fig. 5F–I). The EAMG SVN53-67/M57-KLH treated mice (*n* = 7) demonstrated significantly (*P* = 0.02, t-test) lower mean levels of AChR antibody (0.32±0.14(SD), OD at 450 nm) compared to the mean level of AChR antibody in control EAMG PBS treated mice (*n* = 7; 0.40±0.04(SD), OD at 450 nm) (Fig. 5F). We assessed the IgG subtypes to determine the expression level specific to AChR epitopes. The AChR specific IgG, IgG2a and IgG2b were also lower (*P*<0.02, t-test) (Fig. 5G–I) compared to PBS treated controls. (Table S2).

### AChR levels at the NMJ were increased in treated mice

To determine the effect of reduced AChR antibody levels of mice treated with anti-survivin vaccination, we analyzed the AChR present at the NMJ by labeling with Alexa Fluor 594 -bungarotoxin (Fig. 5J–L) [35] [38] [39]. The AChR at the NMJs of diaphragm in SVN53-67/M57-KLH treated mice (32.39±6.30(SD) pixel density) showed a statistically significant (*P*<0.001, Mann-Whitney U) increase by density scan analysis compared to PBS control mice (23.52±4.52(SD) pixel density). (Table S2)

### Survivin vaccination is protective in active induced experimentally acquired myasthenia gravis in rats

**Figure 6:**
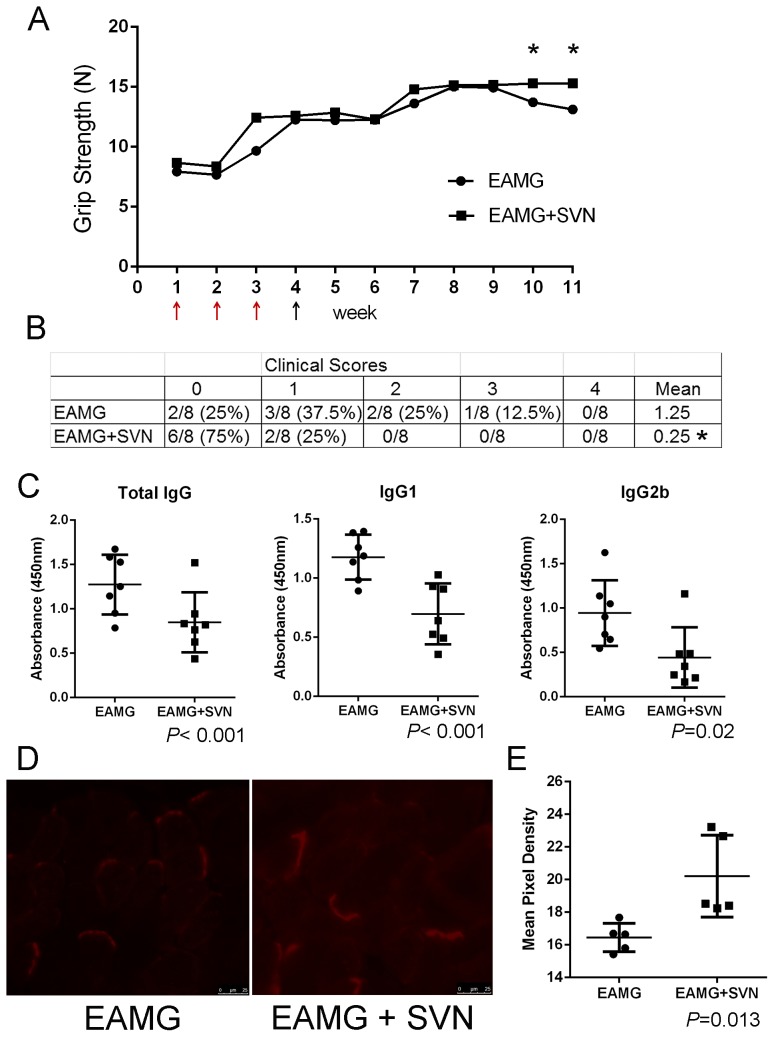
Therapeutic effect of pre-treatment of SVN53-67/M57-KLH on active EAMG. Rats were pre-treated with SVN53-67/M57-KLH (circles) or PBS (squares). **A**) Animals were injected with SVN53-67/M57-KLH at times marked by red arrows. Black arrows mark injection time of tAChR. Grip strength was measured throughout experiment and values are a mean of biweekly measurements. (* denotes *P*<0.02, t-test) **B**) Table of clinical scores based on rats at day 47. **C**) Levels of total IgG, IgG1 and IgG2b were significantly higher in the PBS control group (*n* = 7) compared to SVN53-67/M57-KLH treated animals (*n* = 7). (*P*<0.02 for all, t-test) **D**) SVN53-67/M57-KLH treated rats demonstrated statistically increased amount of AChR (red) at the NMJs compared to PBS-treated rats. **E**) Pixel density of the labeled AChR by Alexa Fluor 594 -bungarotoxin at the NMJ in SVN53-67/M57-KLH treated animals (*n* = 5) compared to PBS treated controls (*n* = 5) (*P* = 0.013, t-test).

We reproduced the effect of SVN53-67/M57-KLH that had been observed in mice by using a second animal model of EAMG. Rats were treated with survivin peptide for three weeks prior to AChR injection to ensure the vaccine had adequate time to induce the production of survivin-specific cytotoxic T lymphocytes (CTL). Pre-treatment with SVN53-67/M57-KLH led to maintenance of grip strength in rats (Fig. 6A, Table S3). Blinded evaluation also revealed significantly (*P*<0.05, t-test) better strength in the treated group with mean clinical scores of 0.25±.46(SD) (*n* = 8) compared to greater weakness in the PBS control group 1.25±1.0(SD)(*n* = 8) (Fig. 6B).

### Reduction in the AChR-specific antibody profile is observed in SVN53-67/M57-KLH treated EAMG rats compared to PBS controls

ELISAs were performed to determine AChR-specific total IgG, IgG1 and IgG2b (Fig.5C, Table S3). As with findings in the mouse model, the EAMG induced rats had reduced expression of AChR antibodies when treated with SVN53-67/M57-KLH. AChR specific-total IgG in the PBS-treated group (*n* = 7, 0.66±0.13(SD), OD at 450 nm) demonstrated higher levels (*P*<0.001, t-test) compared to SVN53-67/M57-KLH treated rats (*n* = 7, 0.46±0.14(SD), OD at 450 nm). The expression levels of IgG1 and IgG2b (1.18±0.19(SD) and 1.26±0.26(SD), OD at 450 nm, respectively) in the PBS control group were statistically higher (*P*<0.02) compared to SVN53-67/M57-KLH treated rats (0.70±0.33(SD) and 0.83±0.37(SD), respectively).

### Analysis of the NMJ demonstrates increased AChR in rats vaccinated against survivin

Animals were sacrificed 47 days after AChR injection. AChR expresssion at the NMJs from diaphragms of rats that had been pre-treated with SVN53-67/M57-KLH (20.53±4.04(SD) pixel density) showed significantly (*P* = 0.013) increased NMJ density with fluorescently labeled bungarotoxin (Fig. 6D) compared to PBS control (16.26±3.49(SD) pixel density)(Fig. 6E, Table S3).

## Discussion

Survivin-positive cells with B lymphocyte and plasma cell markers are present at significant levels in the circulation of humans with MG, but not in controls. In addition, survivin co-localizes to circulating white blood cells which bind specifically to AChR alpha subunit epitopes. Cells within the germinal centers of the thymus of myasthenia gravis patients express survivin. Collectively, these results suggest an association between survivin expression and the autoimmune state in patients with MG. Similarly, rodents with EAMG have large numbers of circulating CD20+/SVN+ (mice) and CD45ra+/SVN+ (rats; data not shown) cells suggesting that, despite fundamental differences in disease induction between MG and EAMG, survivin expression is a common feature of the human disease and these two animal models. Thus, it appears that survivin could be a marker for specific B lymphocytes and plasma cells that are involved in MG and that it may play a key role in enabling cells to resist apoptotic signals leading to sustained pathologic autoantibody production. Reports of survivin expression in lymphoid cells of patients with MS and synovial cells in RA patients suggest that it could play a similar role in other autoimmune diseases as well.

SVN53-67/M57-KLH vaccine attenuated EAMG disease severity as evidenced by improved clinical motor scores, decreased numbers of survivin-expressing CD20+ cells, reduced AChR specific antibodies, reduced antigen-specific complement-fixing antibodies, and moderation of complement deposition. The SVN53-67/M57-KLH was used as a pre-treatment in the rat model and a post-treatment in the mouse model, each demonstrating an improvement in clinical scores. The AChR specific antibody levels of the treated animals were significantly reduced in all IgG subtypes assessed.

Our data suggest that survivin is a factor in the maintenance of autoreactive lymphocytes in spontaneous human disease and in induced models of the disease in rodents. Antibody-mediated autoimmunity is restricted either by modification of the specificity of autoantibodies, transition of the autoreactive B cells to a state of anergy or by the apoptotic deletion of cells that express such autoantibodies [40]. Our study of survivin vaccine in EAMG does not determine the mechanism of action but the results clearly show a reduction in the autoantibodies produced.

Survivin expression has been found in synovial cells from patients with RA and its expression is associated with progression of that disease to a phase involving erosive joint destruction. Similarly, survivin is expressed in PBMC and in the brains of patients with progressive multiple sclerosis. Together with our findings in MG, these observations suggest that survivin plays a pathophysiologic role in some autoimmune diseases by inhibiting the apoptotic cascade that leads to the death of cells that mediate the underlying conditions. Within the limited data set presented, there is a suggestion that survivin-expression in subset of PBMC could serve as a severity and treatment responsive marker, but extensive investigation will be required to confirm such a possibility.

Serum B lymphocyte activating factor (BAFF) levels are increased in patients with autoimmune MG indicating that BAFF is involved in promoting the survival and maturation of autoreactive B cells in the disease [41]. Moreover, CD138-positive plasma cells are abundant in the thymus of MG patients with expression of BAFF and a proliferation-inducing ligand (APRIL) documented in these loci, further suggesting the presence of an environment that is conducive to B-cell survival [42]. In addition, studies have demonstrated that autoantibody-producing plasma cells in the thymus of MG patients are long-lived [43], suggesting an inherent resistance to normal apoptotic signals. The sustained expression of survivin in plasma and B cells of MG patients is consistent with this possibility and provides strong evidence that maintenance of autoimmune cell viability is an important component of the immunopathologic state.

Survivin is a specific and widely expressed cancer protein that is of great interest as a target both for immunotherapy and for specific inhibitors. Survivin targeted treatment approaches using small molecules, RNAi, oligonucleotides, and ribozymes are at various stages of development [44]. SVN53-67/M57-KLH (SurVaxM) is currently being evaluated in Phase I clinical trials as a cancer vaccine [23]. It induces the production of specific anti-survivin antibodies and survivin-reactive T cells by tetramer and ELISPOT analysis in humans (our unpublished data). In addition, studies of human tissues demonstrate that the peptide mimic contained in SVN53-67/M57-KLH is capable of inducing a cytotoxic T cell response capable of killing survivin-expressing B-cell lymphoma cells *ex vivo* [21]. Together with animal data presented here, results suggest that active specific vaccination against survivin might be effective at reducing survivin-expressing autoreactive cells.

Specific survivin inhibitors have been synthesized and at least one of them (YM155) is currently in clinical trials as a cancer therapeutic. Such inhibitors could potentially be useful if survivin levels remain high following clinical immunosuppression; however, the advantage of anti-survivin vaccine therapy rests with its potential to create an extended period of immunity through production of a memory T-cell response, as opposed to life-long medical therapy. In addition, non-specific immunosuppression has many side-effects and risks which may not be the case for active specific vaccination. Previous or concomitant immunosuppressive therapies that are commonly given to MG patients may limit the effect of anti-survivin vaccination in the clinical setting, unless a vaccine is given as initial therapy, or at least early in the course of the illness.

## Supporting Information

Table S1 **Survivin expression in human PBMCs.** Percent of PBMCs from patients with myasthenia gravis and controls expressing survivin; survivin and CD20; survivin and CD27; survivin and 38; survivin and CD138; and survivin and CD8.
(TIF)

Table S2 **Mouse EAMG data.** Numerical values of grip strength, clinical scores, tAChR specific total IgG, tAChR specific IgG1, tAChR specific IgG2a, tAChR specific IgG2b, and pixel density values from the EAMG mouse model.
(TIF)

Table S3 **Rat EAMG data.** Numerical values of grip strength, clinical scores, tAChR specific total IgG, tAChR specific IgG1, tAChR specific IgG2b, and pixel density values from the EAMG rat model.
(TIF)
