# Supplementary figures and images for: Survivin as a Potential Mediator to Support Autoreactive Cell Survival in Myasthenia Gravis: A Human and Animal Model Study
Source: PLoS One. 2014 Jul 22;9(7):e102231. doi: 10.1371/journal.pone.0102231 (PMC4106794; doi:10.1371/journal.pone.0102231)

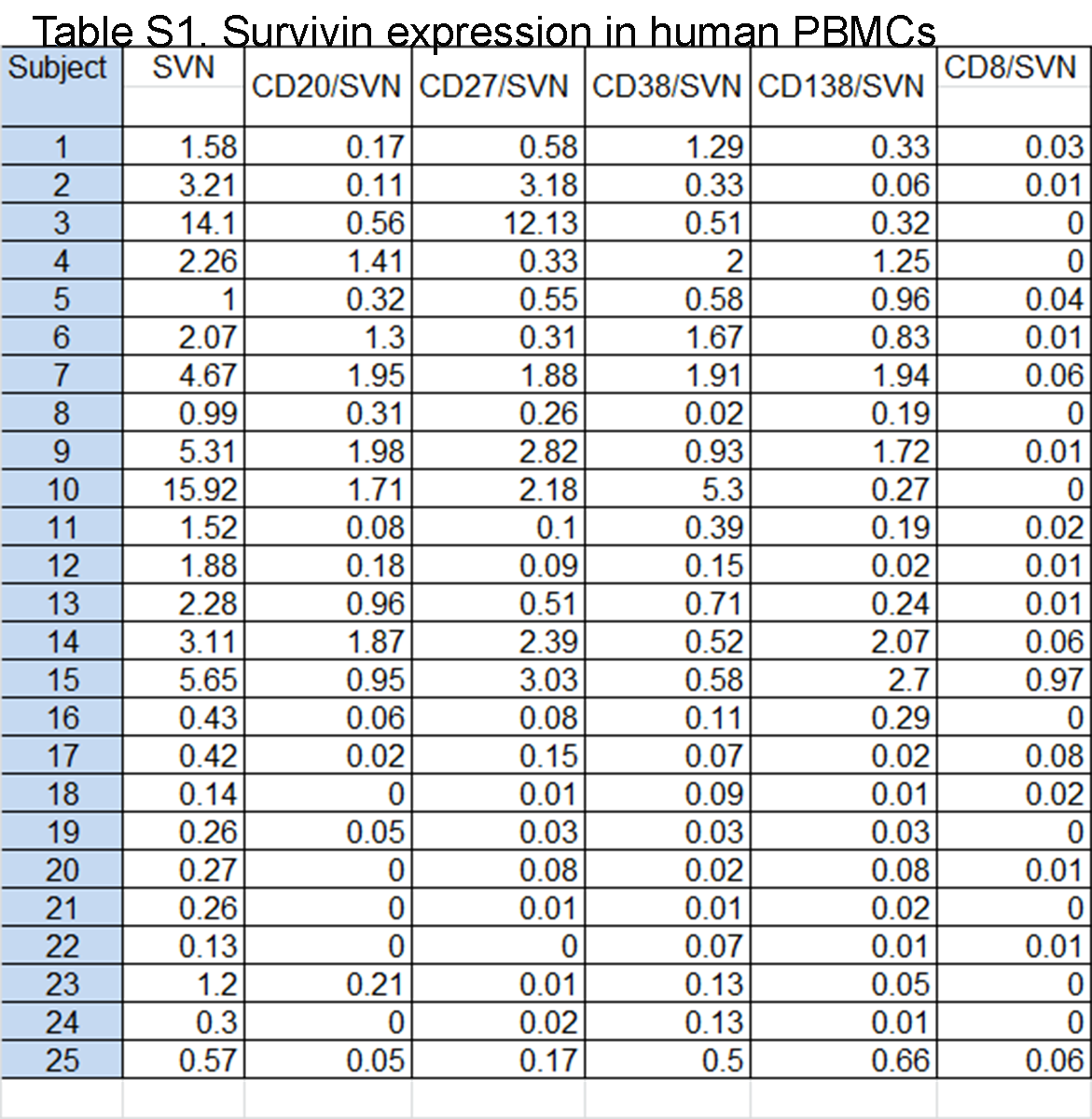

Supplement: Table S1 — Survivin expression in human PBMCs. Percent of PBMCs from patients with myasthenia gravis and controls expressing survivin; survivin and CD20; survivin and CD27; survivin and 38; survivin and CD138; and survivin and CD8. (TIF) [file pone.0102231.s001.tif]

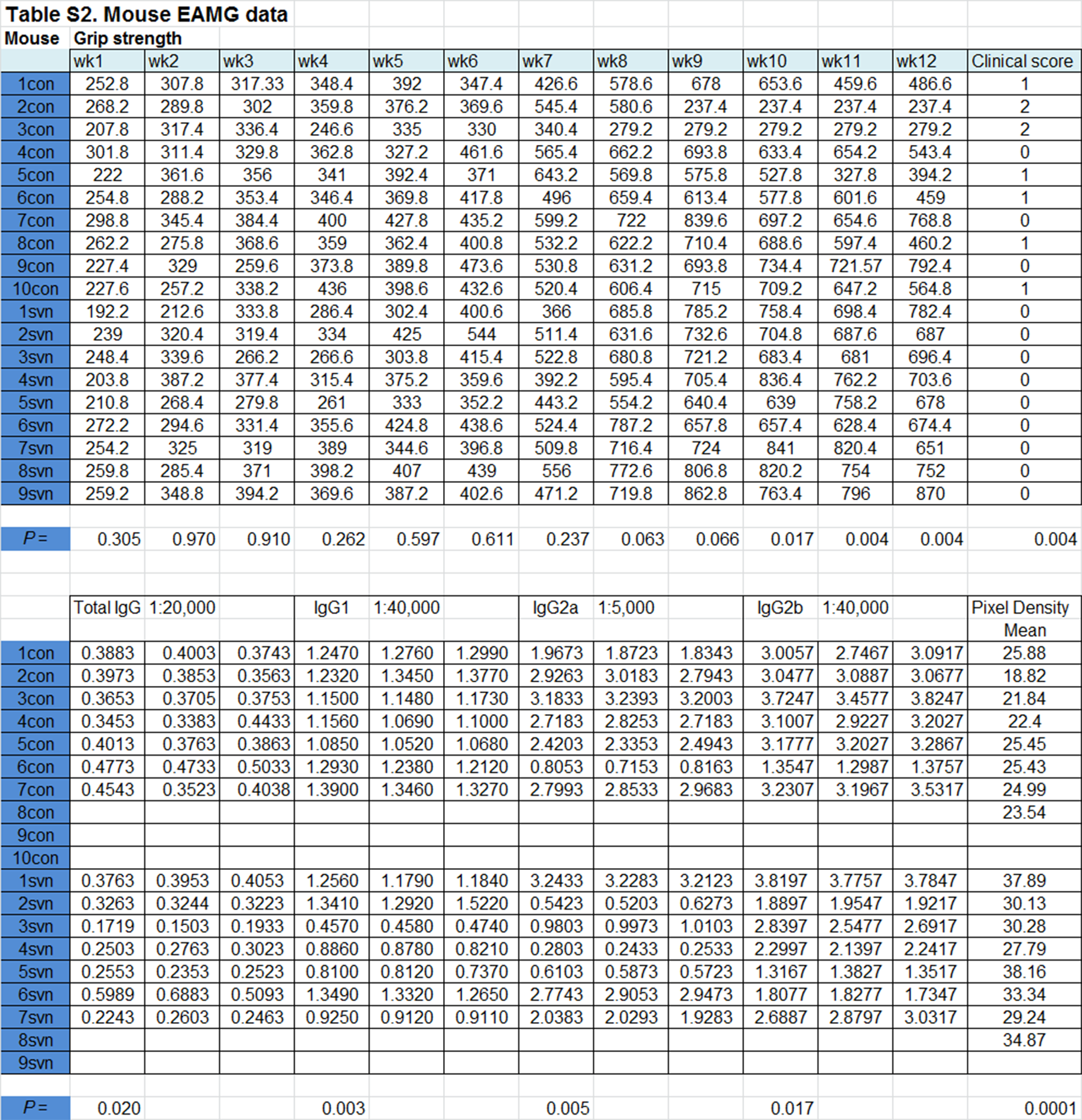

Supplement: Table S2 — Mouse EAMG data. Numerical values of grip strength, clinical scores, tAChR specific total IgG, tAChR specific IgG1, tAChR specific IgG2a, tAChR specific IgG2b, and pixel density values from the EAMG mouse model. (TIF) [file pone.0102231.s002.tif]

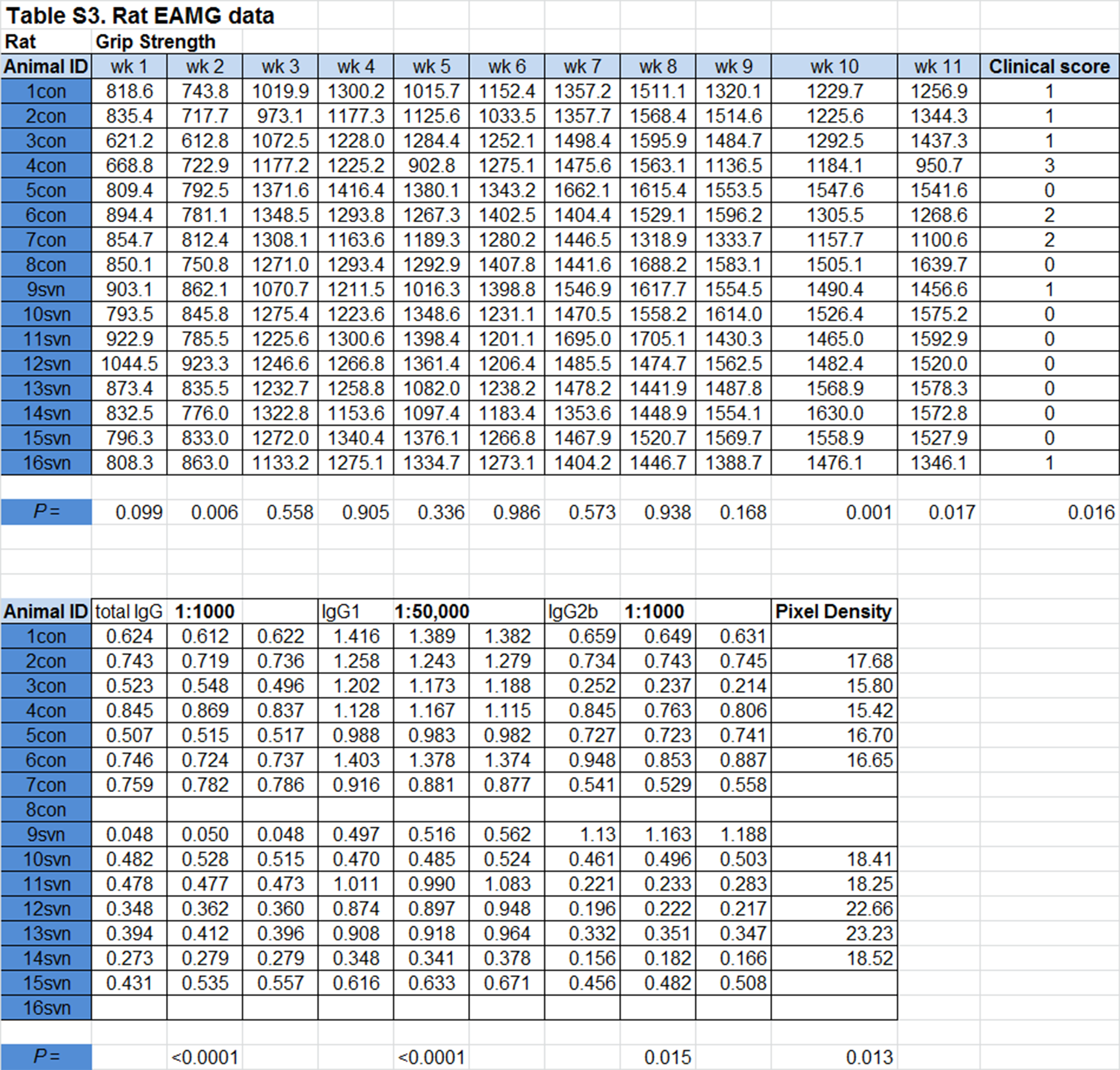

Supplement: Table S3 — Rat EAMG data. Numerical values of grip strength, clinical scores, tAChR specific total IgG, tAChR specific IgG1, tAChR specific IgG2b, and pixel density values from the EAMG rat model. (TIF) [file pone.0102231.s003.tif]
